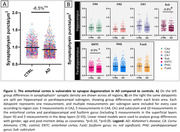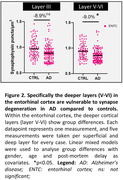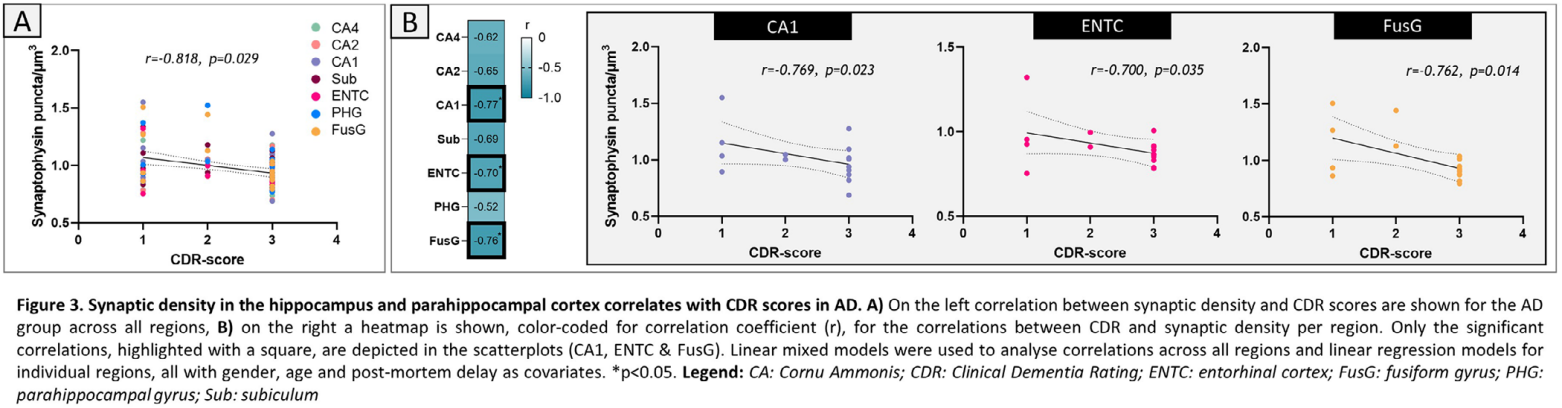# Synaptic density in the hippocampal and parahippocampal subregions and its association with the severity of axonal damage and cognitive decline in Alzheimer’s disease

**DOI:** 10.1002/alz.092170

**Published:** 2025-01-03

**Authors:** Maud M.A. Bouwman, Irene Frigerio, Niels Reijner, Wilma D.J. van de Berg, Laura E. Jonkman

**Affiliations:** ^1^ Amsterdam Neuroscience, Brain Imaging, Amsterdam Netherlands; ^2^ Amsterdam UMC, location VUmc, Department of Anatomy and Neurosciences, Section Clinical Neuroanatomy and Biobanking, Amsterdam Netherlands; ^3^ Amsterdam Neuroscience, Neurodegeneration, Amsterdam Netherlands

## Abstract

**Background:**

The hippocampus is highly vulnerable to amyloid‐b (Aβ) and phosphorylated tau (p‐tau), and shows synaptic loss in Alzheimer’s disease (AD). Moreover, the loss of synapses correlates strongly with cognitive decline and leads to neuronal network dysfunction. Here, we aim to map the selective synaptic loss in hippocampal and parahippocampal subregions in AD and its association to the severity of neuropathology, axonal damage and cognitive decline.

**Method:**

We included (para)hippocampal tissue of 26 AD and 17 age‐matched control donors. Synaptophysin puncta was measured using multiple measurements per subregion, distinguishing superficial and deep cortical layers in the parahippocampus. Aβ and p‐tau load, and NfL immunoreactivity were quantified in manually segmented hippocampal and parahippocampal subregions. Clinical Dementia Rating (CDR) scores were retrieved from the clinical reports. Group differences were analysed using linear mixed models and correlations between synaptic and pathological measures and cognitive scores were studied using linear regression, correcting for age, sex and post‐mortem delay.

**Result:**

The AD (para)hippocampus tended to have a lower synaptic density compared to controls (‐5.6%, p = 0.270). Within hippocampal and parahippocampal subregions, we observed synaptic loss in the entorhinal cortex in AD vs controls (‐8.9%, p = 0.028) and a trend for the subiculum (‐8.8%, p = 0.069) (Fig. 1). Synaptic degeneration was most pronounced in the deeper layers of the enthorinal cortex (layer V‐VI) (Fig. 2). Synaptic loss was not correlated with Aβ and p‐tau load, however higher NfL immunoreactivity associated with synapse loss in the fusiform gyrus (r = ‐0.467, p = 0.008). Synaptic degeneration in the CA1 (r = ‐0.769, p = 0.023), entorhinal cortex (r = ‐0.700, p = 0.035) and fusiform gyrus (r = ‐0.762, p = 0.014) was strongly correlated with higher CDR scores (Fig. 3).

**Conclusion:**

The entorhinal cortex is vulnerable to synapse degeneration in AD. Moreover, our results confirm the strong relation between (para)hippocampal synaptic loss, axonal damage and cognitive decline in AD, while neuropathological load (Aβ and p‐tau) could not explain differences in vulnerability across donors. These results suggest that the synaptic integrity in these regions seem to be important for cognitive function. A better understanding of the selective vulnerability of hippocampal subregions would provide insight into their intricate connections and functions, underpunning their involvement in cognitive decline.